# From efficacy to equity: Literature review of decision criteria for resource allocation and healthcare decisionmaking

**DOI:** 10.1186/1478-7547-10-9

**Published:** 2012-07-18

**Authors:** Lalla Aïda Guindo, Monika Wagner, Rob Baltussen, Donna Rindress, Janine van Til, Paul Kind, Mireille M Goetghebeur

**Affiliations:** 1 BioMedCom Consultants, Montreal, Quebec, Canada; 2 Department of Primary and Community Care, Radboud University Nijmegen Medical Centre, Nijmegen, The Netherlands; 3 University of Twente, Enschede, The Netherlands; 4 York University, Toronto, ON, Canada; 5 Department of Health Administration, Faculty of medicine, University of Montreal, Montreal, Canada

**Keywords:** Decisionmaking, Resource allocation, Priority-setting, Criteria, Healthcare

## Abstract

**Objectives:**

Resource allocation is a challenging issue faced by health policy decisionmakers requiring careful consideration of many factors. Objectives of this study were to identify decision criteria and their frequency reported in the literature on healthcare decisionmaking.

**Method:**

An extensive literature search was performed in Medline and EMBASE to identify articles reporting healthcare decision criteria. Studies conducted with decisionmakers (e.g., focus groups, surveys, interviews), conceptual and review articles and articles describing multicriteria tools were included. Criteria were extracted, organized using a classification system derived from the EVIDEM framework and applying multicriteria decision analysis (MCDA) principles, and the frequency of their occurrence was measured.

**Results:**

Out of 3146 records identified, 2790 were excluded. Out of 356 articles assessed for eligibility, 40 studies included. Criteria were identified from studies performed in several regions of the world involving decisionmakers at micro, meso and macro levels of decision and from studies reporting on multicriteria tools. Large variations in terminology used to define criteria were observed and 360 different terms were identified. These were assigned to 58 criteria which were classified in 9 different categories including: health outcomes; types of benefit; disease impact; therapeutic context; economic impact; quality of evidence; implementation complexity; priority, fairness and ethics; and overall context. The most frequently mentioned criteria were: equity/fairness (32 times), efficacy/effectiveness (29), stakeholder interests and pressures (28), cost-effectiveness (23), strength of evidence (20), safety (19), mission and mandate of health system (19), organizational requirements and capacity (17), patient-reported outcomes (17) and need (16).

**Conclusion:**

This study highlights the importance of considering both normative and feasibility criteria for fair allocation of resources and optimized decisionmaking for coverage and use of healthcare interventions. This analysis provides a foundation to develop a questionnaire for an international survey of decisionmakers on criteria and their relative importance. The ultimate objective is to develop sound multicriteria approaches to enlighten healthcare decisionmaking and priority-setting.

## Review

### Introduction

Resource allocation and priority setting are challenging issues faced by health policy decisionmakers requiring careful consideration of many factors, including objective (e.g., reason) and subjective (e.g., empathy) elements [1]. Criteria used to evaluate healthcare interventions and allocate resources are likely to have profound implications, especially regarding ethical aspects. Ethical principles of resource allocation set forth by the World Health Organization (WHO) include efficiency (maximizing population health), fairness (minimizing health differences) and utility (greatest good for the greatest number) [2]. Consideration of these often conflicting principles requires pragmatic frameworks and the engagement of a broad range of stakeholders to provide accountability for reasonableness (A4R) [3-7]. Limited resources and inequities in healthcare in both wealthy and developing countries underline the need to allocate optimally [8].

As argued by various authors [9-12], choices may not be based on rational and transparent processes highlighting the need for processes that take this into account. Indeed, if the mechanism employed to guide the distribution of resources is inequitable, the outcome is also likely to be. Thus, how resources are allocated by health policy decisionmakers around the world remains a challenging issue [13]. Priority-setting is defined as the process by which healthcare resources are allocated among competing programs or people [14]. In the context of increasing healthcare costs in many countries around the world, effective approaches to explicit appraisal and priority setting are becoming critical to allocate resources to healthcare interventions that provide the most benefit to patient health as well as contributing to healthcare systems’ sustainability, equity and efficiency. Indeed, elucidating decision criteria and how they are considered are key to establishing accountability and reasonableness of decisions and fulfils the A4R framework set forth by Daniels and Sabin [6].

Over the past decades, a number of empirical studies have explored systematic approaches to optimize evaluation of healthcare interventions and priority-setting. A number of tools with defined criteria to evaluate and rank interventions have been developed, recognizing the need for such approaches [10,15-28]. As part of a larger collaborative endeavour exploring decision criteria, the aim of this study was to analyse the peer-reviewed literature to identify criteria reported in empirical studies that involved healthcare decisionmakers and in studies describing multicriteria tools. The specific objectives were to identify, categorize and estimate the frequency of decision criteria reported in the literature. This work will support the design of an international survey of decisionmakers on criteria and their relative importance as well as providing a resource for developers of multicriteria-based frameworks.

### Methods

#### Search strategy and article selection

An extensive literature search was carried out in June 2010 on Medline and EMBASE databases to identify articles reporting healthcare decision criteria. Because studies reporting criteria (or factors or principles or components) are usually not indexed with such generic terms and because these terms are used in many fields (e.g., diagnostic criteria), a number of algorithms were explored to optimize the search strategy. The optimized search strategy included the following keywords: “decision-making”, “priority-setting”, and “resource allocation”, combined with “funding”, “budget”, “cost-benefit analysis”, “cost-effectiveness analysis”, and “equity”. The research was limited to articles published in English, French, or German over the last 10 years and excluded the following types of studies: clinical trials (phase I to IV), editorials, letters, randomized controlled trials, case reports, and comparative studies. Bibliographies of relevant articles were also searched.

Abstracts of articles thus retrieved were screened to identify appropriate inclusion and exclusion criteria. Studies were included if they reported a set (i.e., > 1) of decision criteria and were:

empirical studies conducted with healthcare decisionmakers (including field-testing of decisionmaking tools, focus groups, questionnaires, interviews)

reviews of such empirical studies, and

conceptual studies describing or proposing a set of decision criteria or a decisionmaking tool.

Studies were excluded if they focused on a single criterion (e.g., cost-effectiveness only) or described a priority-setting exercise without explicitly identifying decision criteria. Studies discussing the goals and advantages of priority-setting *per se* without reporting specific criteria were also excluded. To avoid double-counting of decision criteria, only one publication was included if several publications from the same group described the same set of decision criteria. For the same reason, studies reported in review articles that we included in our analysis and which reported the criteria of the original studies were also excluded.

#### Data extraction

Full texts of selected articles were reviewed and data extracted into a table identifying: 1) first author; 2) year of publication 3) method of criteria elicitation or identification, 4) decisionmaking setting, 5) exact term for each criterion as reported in the publication.

**Figure 1:**
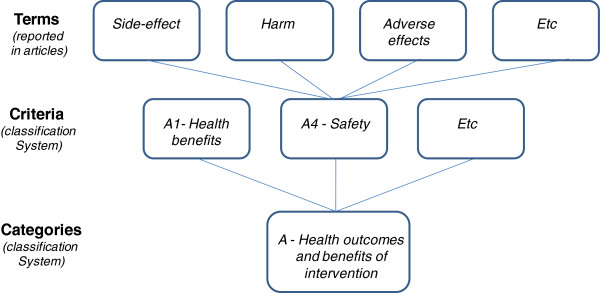
** Categorization of terms reported in the literature**.

Given the variability of terms to describe conceptually similar decision criteria, a hierarchical classification system was developed (Figure 1). Terms referring to the same concept (e.g., “side-effects” and “harm”) were grouped under one criterion (e.g., Safety). Related criteria were grouped under categories (e.g., Health outcomes and benefits of intervention). This process of classification was guided by the structure of the EVIDEM framework, which includes an adaptable set of core and contextual criteria identified from analyses of the literature, of decisionmaking processes worldwide, and discussions with decisionmakers, and which were structured to fulfill the requirements of multicriteria decision analysis (MCDA; i.e., minimum overlap, mutual independence, operationalizability, completeness and clustering) [10,18,29]. MCDA principles were applied in the present study to define criteria regrouping terms referring to the same concept and to categorize criteria into a meaningful and intuitive architecture (clustering). 

#### Descriptive statistics

The number of times each criterion was cited in the studies retrieved was used as a proxy to identify the criteria perceived to be most important. Descriptive statistics were performed and each occurrence of a term belonging to that criterion was counted. If a study reported two different terms that we grouped under the same criterion, both terms were counted. For example, if a study reported “side effects” and “harm” as separate terms, we counted both of them under the criterion “Safety”. The numbers of citations for each criterion and for each category of criteria were analyzed.

### Results

#### Identification of decision criteria from the literature review

**Figure 2:**
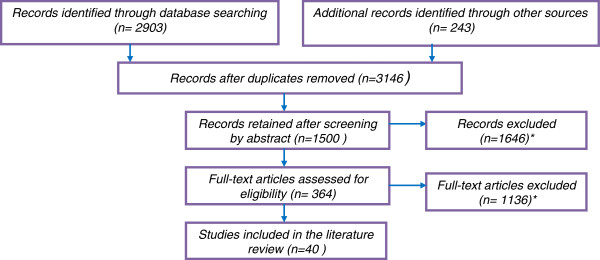
PRISMA diagram.

The literature search resulted in a total of 2903 records identified through PUBMED and EMBASE database searching and 243 additional records were identified through bibliographic hand searching (Figure 2). These studies were screened by their abstracts and 2790 were excluded. The remaining 364 studies were assessed for eligibility on the basis of full text and 317 articles were excluded. A total of 40 studies were included (Table 1), all of which were published after 1997, and 33 studies from 2006 to 2010. The majority of studies reported criteria derived from interviews and focus groups (9 studies each) surveys (2) or literature review of studies (5) conducted with healthcare decisionmakers at micro, meso and macro levels of decision and from several regions of the world. Fourteen studies described multicriteria decisionmaking tools.

**Table 1:** Studies identified in the literature and included in the analysis

**Studies reporting on decision criteria**		**Studies describing a decisionmaking tool**
**Authors**	Type of study and level of decisionmaking*	
1. Andreae et al. [9], 2009	Survey, macro	1. Bowen et al. [15], 2005
2. Asante et al. [8], 2009	Interviews, meso & macro	2. Browman et al. [16], 2008
3. Baltussen et al. [12], 2007	Focus group, macro	3. Ghaffar et al. [17], 2010
4. Baltussen et al. [30], 2006	Focus group, meso & macro	4. Goetghebeur et al. [10,18], 2008,2010
5. Baltussen et al. [11], 2006	Methodology	5. Golan et al. [19], 2010
6. Dionne et al. [31], 2009	Interviews, macro	6. Hailey et al. [20], 2009
7. Dolan et al. [32], 2010	Methodology	7. Honore et al. [21], 2010
8. Duthie et al. [33], 1997	Interviews, micro, meso & macro	8. Johnson et al. [22], 2009
9. Gibson et al. [34], 2006	Focus group & interviews, meso & macro	9. Kirby et al. [23], 2008
10. Hofmann et al. [35], 2005	Literature review	10. Meagher et al. [24], 2010
11. Irving et al. [36], 2010	Interviews, micro	11. Menon et al. [25], 2010
12. Jehu-Appiah et al. [37], 2008	Focus group, macro	12. Tannahill et al. [26], 2008
13. Kapiriri et al. [38], 2009	Interviews, micro, meso & macro	13. The University of York [27], 2002
14. Koopmanschap et al. [39], 2010	Focus group, macro	14. Wilson et al. [28], 2006
15. Lasry et al. [14], 2010	Interviews, macro	
16. Lehoux et al. [40], 2007	Literature review	
17. Lopert et al. [41], 2009	Focus group, macro	
18. Martin et al. [42], 2001	Focus group, macro	
19. Mitton et al. [43], 2006	Focus group, macro	
20. Mullen et al. [44], 2004	Survey, meso	
21. Noorani et al. [45], 2007	Literature review and interviews, macro	
22. Saarni et al. [46], 2008	Consensus procedure, macro	
23. Vuorenkoski et al. [47], 2008	Literature review	
24. Wilson et al. [48], 2007	Focus group, macro	
25. Wirtz et al. [49], 2005	Interviews, macro	
26. Youngkong et al. [13], 2009	Literature review	

*Survey, interviews and focus groups were performed with healthcare decisionmakers making decisions at national or regional level (macro level), at a healthcare facility level (meso level) and/or at the healthcare provider level (micro level).

#### Decision criteria classification and descriptive statistics

Large variations in terminology used to define criteria were observed among the studies included; 360 different terms were identified (Table 2). Using the classification system described above, these terms were assigned to 58 unique criteria which were classified into 9 different categories. These were: A) health outcomes and benefits of intervention (6 criteria), B) types of health benefit (2 criteria), C) impact of disease targeted by intervention (4 criteria), D) therapeutic context of intervention (4 criteria), E) economic impact of intervention (9 criteria), F) quality/uncertainty of evidence (6 criteria), G) implementation complexity of intervention (9 criteria), H) priorities, fairness and ethics (7 criteria), I) overall context (11 criteria). Categories were defined to: i) regroup criteria pertaining to the same overall concept (e.g., category “A - Health outcomes and benefits” of intervention includes criteria such as health benefits, life saving, efficacy, effectiveness, safety, patient-reported outcomes and quality of care) and to ii) disentangle criteria specific to the intervention (categories A to F) from criteria specific to the context (G to I).

**Table 2:** Classification of terms reported in the literature

**Categories of classification system**	**Criteria of classification system**	**Terms used in articles**
**A-Health outcomes and benefits of intervention**	Number of criteria: 6	Number of terms: 44
	A1: Health benefits: 7 terms, cited 10 times	· A1 – health benefits [13,31,38,50], potential health gain [44], enhanced health outcomes [44], relative advantage [51], health effects [30], additional effects [22], incremental health gain [43]
	A2: Efficacy/effectiveness: 11 terms, cited 29 times	· A2 – efficacy [13,47], efficacy/effectiveness [10,19,20,25,27,28,44,48], effectiveness [14,22,26,32-34,48], clinical benefit [19,22,24,42,47], clinical impact [45], clinical merit [22], relative clinical benefit in relation with current standards [16], determine relative value for degree of benefit against benchmarks [16], magnitude of treatment effect [22], response rate [43], onset and duration of treatment/program effect [43]
	A3: Life saving: 4 terms, cited 5 times	· A3 – prolongation of disease-free survival [42], saving life [19], life expectancy gains [13], average life-year benefit per patient [13,33]
	A4: Safety: 11 terms, 19 times	· A4 – side effects [33,41,47], unintended consequences [40], safety [9,22,26,31], safety and tolerability [10,19,20], risks [20,22], risk management [44], harm [42], adverse effects [32], inconvenience [22], risk of event [22], reduction in symptomatic toxicity compared with standard therapy [42]
	A5: PRO: 10 terms, 17 times	· A5 – patients reported outcomes [10], quality of life [19,42,44,52], impact on quality of life [22,43], number of QALYs gained per patient [36,39], disability adjusted life years [13], likely impact on patient [16], patient preference [25], patient autonomy [26,35,40], relative value to patient [16], best for patient [38]
	A6: Quality of care: 1 term, 1 time	· A6 – overall gain in quality of care [44]
**B-Type of health benefit**	Number of criteria: 2	Number of terms: 12
	B1: Population effect (prevention): 6 terms, 11 times	· B1 – public health interest [10], population effects [19], prevention [19,28], prevention of ill health [44], social impact [13,22,33], social benefit [13,22,33]
	B2: Individual effect (medical service): 6 terms, 7 times	· B2 – type of medical service [10], relief/prevention of symptoms/complications of disease [42], health gain or maintenance [44], individual effects [19], individual impact and benefit [13,33], the composition of the health gain [39]
**C-Impact of the disease targeted by intervention**	Number of criteria: 4	Number of terms: 21
	C1: Disease severity: 2 terms, 9 times	· C1 – severity of disease [9,10,13,19,30,37,39,47], impact of the disease/condition on quality of life [43]
	C2: Disease determinants: 2 terms, 2 times	· C2 – determinants (the factors responsible for the persistence of the burden) [17], characteristics of target condition [22]
	C3: Disease burden: 7 terms, 13 times	· C3 – burden of disease [9,13,22,33], disease burden [17,25,45,48], burden of illness [22], burden of therapy [22], cost to treat disease [33], cost to prevent disease [33], national cost of the disease/condition to the healthcare system [43]
	C4: Epidemiology: 10 terms, 16 times	· C4 – prevalence [9,13], number of potential beneficiaries [35,37,40], indirect beneficiaries [40], size of population [10,19], prevalence and incidence of disease [23,25,43], number of residents benefiting [44], number of clients served [43], number of patients [47], social/demographics [22], incidence [22]
**D-Therapeutic context of intervention**	Number of criteria: 4	Number of terms: 18
	D1: Treatment alternatives: 5 terms, 13 times	· D1 – treatment alternatives [13,22], availability of alternatives [16,19,25,42,44,47], availability of effective intervention and preventable [13], alternatives [35,40,45], benchmark comparators [16]
	D2: Need: 8 terms, 16 times	· D2 – comparative interventions limitations (unmet needs) [10], need [19,22,28,38,42,44,49], clinical impact (need and trends) [24], emergencies and need [13], apparent need [14], clinical need [36,41,50], desirability of effects [40], meets patient’s basic need [38]
	D3: Clinical guidelines & practices: 4 terms, 7 times	· D3 – evidence-based guidelines [13,33,36], best practice [14], clinical guidelines [10,23], academic health center research (establishing/or using best practice) [24]
	D4: Pre-existing use: 1 term, 1 time	· D4 – pre-existing prescribing of the drug [47]
**E-Economic impact of intervention**	Number of criteria: 9	Number of terms: 36
	E1: Cost: 3 terms, 11 times	· E1 – cost per patient [19], costs [19,20,22,27,32,42,44,47,51], unit cost [22]
	E2: Budget impact: 6 terms, 11 times	· E2 – budget impact on health plan [10,19,25,47], total budget impact [30], budget impact [32,45,47], usage and cost implications of competing new drugs if approved [16], affordability [25], operating and start-up costs [43]
	E3: Broad financial impact: 7 terms, 7 times	· E3 – impact on other spending [10], financial impact on government [13], economic impact [45], economics [22], national medical costs per-year [39], cost-saving [33], national saving in costs of absence per year [39]
	E4: Poverty reduction: 1 terms, 3 times	· E4 – positive poverty reduction [13,30,37]
	E5: Cost-effectiveness: 5 terms, 23 times	· E5 – cost-effectiveness [9,10,13,14,17,20,22,25-27,30,34,37,39,41,44], economic evaluations [27], cost and consequences [9,13,14,41], pharmacoeconomic analysis [23], cost utility expressed as cost per QALY [22]
	E6: Value: 2 terms, 3 times	· E6 – value for money [32,44], financial value [44]
	E7: Efficiency and opportunity costs: 6 terms, 10 times	· E7 – efficiency of intervention [31], efficiency [10,19,22,23,44], opportunity costs [10], opportunity costs to the population/society [16], best within available resources [38], interdependencies [50]
	E8: Resources: 5 terms, 6 times	· E8 – resources [17,51], variation in rate of use [45], available resources [13], resources implications [50], volume of activity [13]
	E9: Insurance premiums: 1 term, 1 time	· E9 – impact on health insurance premiums [9]
**F-Quality and uncertainty of evidence**	Number of criteria: 6	Number of terms: 34
	F1: Evidence available: 7 terms, 9 times	· F1 – evidence [22,42,45], proof [22], scientific evidence [47], current level of knowledge [17], time of assessment in technology development [35], timelines of review [45], therapy mechanism of action [23]
	F2: Strength of evidence: 14 terms, 20 times	· F2 – strength of evidence [16,44], quality of evidence [47], quality of data and past decisions [47], quality of data [22], quality [26], validity of evidence [10,19], related degree of knowledge certainty [23], certainty [48], consistency [19,22,44], consistent [38], completeness and consistency of reporting evidence [10], openness [26,44], selection of studies [35,40], precision of treatment effect [22]
	F3: Relevance of evidence: 5 terms, 8 times	· F3 – relevance of evidence [10,19], representativeness of users (studies vs. real world) [35,40], level of generalization [35,40], effectiveness in real practice [22], evidence of effectiveness [44]
	F4: Evidence characteristics: 5 terms, 7 times	· F4 – normative characteristics of study [35,40], choice of endpoints [35,40], clinical trial data [47], multiple randomized trials or meta-analysis/single randomized trial of reasonable size/small randomized trial [42], phase II [53]
	F5: Research ethics: 2 terms, 4 times	· F5 – research ethics [35,40], informed consent [26,40]
	F6: Evidence requirements: 1 term, 1 time	· F6 – adherence to requirement of decision making body [10]
**G-Implementation complexity of intervention**	Number of criteria: 9	Number of terms: 57
	G1: Legislation: 6 terms, 6 times	· G1 – legal arrangements [40], legislative issues [22], medical liability [40], human rights legislation [23], legal implications [45], conformity of programs [22]
	G2: Organizational requirements and capacity to implement: 15 terms, 17 times	· G2 – system requirements [25], physical environment [44], environment [22,26], system capacity [10], local capacity [17], ability to implement [38], implementation [22], organization’s structure [51], organizational burden [49], logistics [36], process [28], well-organized [38], organizational feasibility [22,25], feasibility of delivery [16], deliverability [48]
	G3: Skills: 6 terms, 6 times	· G3 – knowledge and skills [51], nature of staff [51], clinical education and training [44], human resources availability [17], recruitment and retention of staff [44], attracting/retaining scarce clinical staff [44]
	G4: Flexibility of implementation: 7 terms, 8 times	· G4 – flexibility [51], reversibility [51], trialiability [51], revisability [51], ability to evaluate [22], provision for revision/appeals [38], engagement [26,48]
	G5: Characteristics of intervention: 6 terms, 8 times	· G5 – characteristics of intervention [22], complexity of the intervention [51], components of technology [35], autonomy of the intervention [38], autonomy [17,26,46], convenience [42]
	G6: Appropriate use: 3 terms, 3 times	· G6 – appropriate use of intervention [10], appropriateness [44], appropriate setting/level of service [43]
	G7: Barriers and acceptability: 3 terms, 4 times	· G7 – acceptability [22,48], responsiveness [44], controversial nature of proposed technology [45]
	G8: Integration and system efficiencies: 9 terms, 9 times	· G8 – system integration (best use of elements of healthcare system) [34], integration into local community [44], ease of integration [22], impact on other services [40], links to other services [44], compatibility [22], reduction of the monitoring [33], reduction of waiting list size [33], impact [22]
	G9: Sustainability: 2 terms, 4 times	· G9 – sustainability [23,24,26], longevity [19]
**H-Priorities, fairness and ethics**	Number of criteria: 7	Number of terms: 55
	H1 Population priorities: 5 terms, 5 times	· H1 – perspective and current priority [13], target and priority-setting [14], known priorities [44], population priority [10], coverage of selected conditions [13]
	H2 : Access: 10 terms, 17 times	· H2 – population access [10], access [19,27,47,49], equity of access improvement [13], access to care easier [31,33,34], distribution and access to healthcare [35,40], accessibility [22,44], equity of access [44], access to health system [22], geographical equity [43], timeliness of access [43]
	H3 : Vulnerable and needy population: 9 terms, 11 times	· H3 – vulnerable population [37,38], potential victims [40], particular social groups with high risk and/or increased vulnerability [23], compassion for the vulnerable [19], particularly needy/vulnerable groups [44], age of targeted group [13,30], maternal mortality [13], quality of maternity care services [13], population equity [43]
	H4: Equity, fairness and justice: 12 terms, 32 times	· H4 – equity [8,13,14,19,22,25,27,40,44,46,48], fairness [10,14,40,44,47], health equity [23,26], equality [19,26,38], distributive justice [23,25], formal justice [23], social justice [23], justice [26,46], social injustice [40], addressing health status inequalities at a population level [44], human integrity and dignity [35,40], basic human rights [35]
	H5 : Utility: 2 terms, 3 times	· H5 – utility [10,26], utilitarism [25]
	H6: Solidarity: 6 terms, 8 times	· H6 – solidarity [19,25,26], collectivism [26], mutuality [26], reciprocal trust [40], diversity [26], cohesion [26]
	H7: Ethics and moral aspects: 11 terms, 14 times	· H7 – ethics [14,22], ethical values [22], values [22], values and beliefs [51], consistency with societal values [22], ethical implications [45], moral obligation to implement a technology [35,40], rule of rescue [25], priority to basic and necessary care [38], moral consequence of HTA [35,40], moral challenges related to certain components of HTA [35]
**I-Overall context**	Number of criteria: 11	Number of terms: 83
	I1: Mission and mandate of health system: 13 terms, 19 times	· I1 – goals of healthcare [52,53], goals [21], beneficence [28], non-maleficience and justice [28], beneficence/non-maleficience [17,26,53], strategic fit [9,23], medical and social worth [45], relevance [22], present social consensus, [17,49] consensus regarding public funding of a therapy [17,53], government mandate [17], national standards [24], healthcare context positioning [23]
	I2: Overall priorities: 6 terms, 6 times	· I2 – national priorities [45], national or board priority [14], local and national priorities [8], international priorities [45], alignment with external directives [9], strategic direction [43]
	I3: Financial constraints: 8 terms, 13 times	· I3 – budget constraints [13,33,45], cost-containment [42,49], budget level [13,19,45], social economical context [16], limited provincial health resources [17], budget implementation challenges [17], economic feasibility [37], reliance of other services/sectors(on investment) [14]
	I4: Incentives: 4 terms, 5 times	· I4 – financial incentives [28,45], organizational support [16], donor involvement [31], incentives for compliance [20]
	I5: Political aspects: 5 terms, 7 times	· I5 – political pressure [13,19,45], political components [52], politically and legally defensible decisions [42], politics [37], political impact [37]
	I6: Historical aspects: 3 terms, 3 times	· I6 – historical components [52], past experiences [16], historical budgets [19]
	I7: Cultural aspects: 7 terms, 10 times	· I7 – culture and religious convictions [19,28,47], stigma [28], compatibility with values [16], challenge of social and values arrangements [28,47], conception of certain persons or disease [47], psychosocial implications [34], public preference [14]
	I8: Innovation: 3 terms, 3 times	· I8 – perceived benefits of change [16], innovativeness [37], generation or application of knowledge [43]
	I9: Partnership and leadership: 8 terms, 9 times	· I9 – partnership and networking [16], partnerships [9], maintaining relationship [42], leadership [16], community development [53], academic commitments: research and education [9,23], partnership and collaboration across organizations [43], contribution to position as a learning organization [43]
	I10: Citizen involvement: 3 terms, 3 times	· I10 – citizenship [53], ownership [53], enabling health literacy (empowerment) [53]
	I11: Stakeholders interests and pressures: 23 terms, 28 times	· I11-stakeholders pressure [52], advocacy [16,45], pressure from physician and patients groups and past decisions [32], clinical expert opinions [37], patient representative group opinions [37], power relations among stakeholders [28], user of the technology interests [47], challenge the relationship between patient and physician [47], professional prestige [28,47], clinicians excitement and decisions in other hospitals [32], public reaction and public accountability [28], HTA’s producer interest [28,47], company activities [32], researchers ethics interests [28,47], third party agents involved [47], recommendations made by other countries [13], status in other jurisdictions [49], current status of public funding in other jurisdictions [17], drugs used in other hospitals [32], expressed demand [14,37], patient demand [32], expected level of interest (patient and medical) [34], entitlement [28]

This table is reporting all the terms (338) extracted from the selected articles and tabulates them using the classification system developed for this study, which is based on a hierarchical approach clustering 58 criteria into 9 categories.

**Figure 3:**
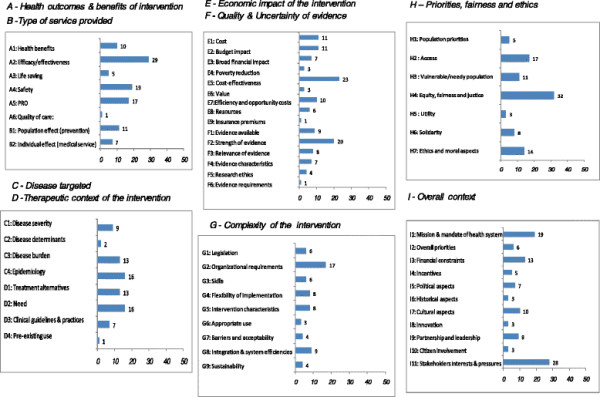
Classification system and number of citations for each criterion.

The classification system and the number of citations for each criterion are reported in Figure 3. The ten most frequently mentioned criteria were: equity, fairness and justice (H4, 32 citations); efficacy/effectiveness (A2, 29 citations); stakeholder interests and pressures (I11, 28 citations); cost-effectiveness (E5, 23 citations); strength of evidence (F2, 20 citations); safety (A4, 19 citations); mission and mandate of health system (I1:19 citations); organizational requirements and capacity (G2, 17 citations); patient-reported outcomes (A5, 17 citations); and need (D2, 16 citations). Among these 10 most frequently cited criteria, three criteria were from the category “A - Health benefits and outcomes of intervention”, highlighting the importance of this consideration in decisionmaking. The other most frequently cited criteria were from seven categories of criteria, indicating that the classification system captured critical criteria in distinct categories.

**Figure 4:**
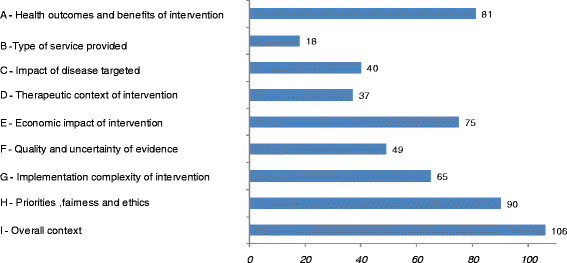
Number of citations for each category of criteria of the classification system.

 At the category level (Figure 4), the number of citations was the highest for the category of criteria “Overall context” (106 citations); followed by “Priorities, fairness and ethics” (90 citations); “Health outcomes and benefits of intervention” (81 citations); “Economic impact of intervention” (75 citations); “Implementation complexity of intervention” (65 citations); “Quality and uncertainty of evidence” (49 citations); “Impact of disease targeted” (40 citations); “Therapeutic context of intervention” (37 citations); and “Type of service provided” (18 citations).

### Discussion

This literature review revealed a burgeoning number of studies examining healthcare decision criteria and criteria-based decisionmaking tools, especially over the last five years. Criteria were identified from studies performed in several regions of the world involving decisionmakers at micro, meso and macro levels of decision and from studies reporting on multicriteria tools. Increasingly, the healthcare community is aware that beyond cost-effectiveness, other criteria must be taken explicitly into account for transparent and consistent healthcare decisionmaking and priority-setting [54-56]. Indeed, elucidating decision criteria and how they are considered are key to establishing accountability and reasonableness of decisions. This is necessary to fulfill the relevance condition of the accountability for reasonableness (A4R) framework of Daniels and Sabin [6], which states that “Decisions should be made on the basis of reasons (i.e. evidence, principles, values, arguments) that ‘fair-minded’ stakeholders can agree are relevant under the circumstances”.

This analysis revealed a predominance of normative criteria, that is, answering the question “what should be done?” This highlights the importance of considering the actual worth or value of healthcare interventions rather than just feasibility criteria, (“What can be done?”). Of the ten most frequently cited criteria, eight were normative (equity and fairness, efficacy, cost-effectiveness, strength of evidence, safety, mission and mandate of healthcare system, need, patient-reported outcomes) and two were feasibility criteria (stakeholder pressures and interests, organizational requirements and capacity). This is aligned with a review of studies on decision criteria in developing countries [13], and points to the need to include both normative and feasibility criteria in decision and prioritization tools to fully reflect and support the decisionmaking process.

The criterion “equity and fairness” was the most frequently reported. This may reflect that equity is a guiding principle in defining the values on which decisions are based. Equity is difficult to operationalize in decisionmaking and priority-setting processes in a pragmatic manner. It is a complex ethical concept that eludes precise definition and is synonymous with social justice and fairness [57]. It is referred to as “a fair chance for all,” [23] “equality of access to healthcare resources on the basis of need,” [8] “absence of systematic disparities in health (or in the major social determinants of health) between groups with different levels of underlying social advantage/disadvantage” [58]. The WHO advocates concepts of “horizontal equity, providing healthcare to all those who have the same health need, and vertical equity, providing preferentially to those with the greatest need” [57]. The difficulty of considering equity in a pragmatic manner points to the need to include it systematically as operationalizable criteria in the decision process. If not systematic, it is less likely that decisions will be equitable. Decisions are generally fairest when standards are predetermined, explicit and consistently applied [59]. Equity is embedded in consideration of disease severity in prioritization of healthcare interventions. Decisionmakers generally attach more value to interventions for severe disease than for mild disease. This is also translated in the worst-off principle, which relates to an independent concern for severity; “the worse off an individual would be without an intervention, the more highly society tends to value that intervention” [60]. Systematic consideration of criteria defined on the basis of population priorities identified by decisionmakers (e.g., more value for interventions targeted to vulnerable populations such as children, the elderly, those in remote areas) is another pragmatic way to incorporate equity into decisionmaking. Integration of ethical considerations in operationalizable criteria was developed for the comprehensive multicriteria framework EVIDEM [61]. Ethical issues are an integral part of the EUnetHTA core model to ensure their explicit considerations [46], and several frameworks focusing on equity [62] and ethical issues [63] have recently emerged.

Efficacy/effectiveness was the second most frequently reported criterion; as Hawkes discussed recently, “governments are wrestling with the issues of efficacy and fairness in healthcare delivery” [64]. While efficacy measures the effect of an intervention treatment under controlled conditions (such as during clinical trials), effectiveness provides critical information on outcomes actually achieved by an intervention in real life settings. Efficacy and effectiveness are fundamental criteria considered at the regulatory (e.g., FDA, EMA) and reimbursement levels for medicines in many jurisdictions [65-67]. Because decisions concerning interventions at policy, clinical and patient level are made with reference to a given context of care (usually standard of care), improvement over existing care rather than absolute efficacy or effectiveness provides the most informative evidence [10]. Indeed, decisions about usefulness of interventions are usually based on relative advantage compared to existing approaches [15]. Comparative effectiveness, “the comparative assessment of interventions in routine practice settings” [68] is meant to help answer the question “does it work in my context?” and is demand-driven research aimed directly at decisionmaker needs [69]. For new interventions, however, effectiveness data is usually not available and decisions are often made on the basis of efficacy data, with the uncertainty inherent in innovation [67]. Evidence-based decisionmaking relies on actual benefits derived from an intervention so mechanisms (such as defining subcriteria) outlining specifically the most relevant outcomes of efficacy/effectiveness in real life are critical to ensure that the dimensions of efficacy/effectiveness are fully captured and communicated.

The third most commonly reported criterion refers to stakeholder interests and pressures. Macro-level decisions are influenced by public pressure and advocacy [13,15,38] and the demand for a new program is a powerful argument for decisionmakers at the political level [70]. In a study exploring the basis for immunization recommendations, while vaccine safety was reported as important or very important in making immunization recommendations by all countries regardless of economic status, low and lower middle income countries were significantly more likely than developed countries to report that public pressure was an important factor [9]. Because pressures from groups of stakeholders are often part of the context [10], being aware of pressures and interests at stake and how they may affect decisionmaking and implementation is important and should be explicitly tackled using a framework that encourage systematic consideration of their potential implications when making healthcare decisions.

Cost-effectiveness was the fourth most commonly reported criterion. Cost-effectiveness is frequently used in healthcare decisionmaking [65,71] but its usefulness is the subject of debate [54,56]. A review of 36 empirical studies reported that the influence of cost-effectiveness was moderate at micro, meso and macro levels of decision [55]. Designed to incorporate several criteria of decision (e.g., cost, efficacy/effectiveness, safety, quality of life) into an aggregated ratio allowing comparisons of interventions, it fails to include important criteria such as equity and the severity of the targeted condition [59]. In addition, cost-effectiveness thresholds are commonly mistaken for affordability thresholds [59]. Beyond cost-effectiveness ratios, health economic studies generate data that are necessary to evaluate healthcare interventions (e.g., resource utilization and cost consequences of a new intervention compared to existing care).

This study also revealed that strength of evidence is an important aspect in decisionmaking, highlighting the influence of evidence-based medicine. Evidence is usually sought to demonstrate effectiveness (“it works”), show the need for policy action (“it solves a problem”), guide effective implementation (“it can be done”), and clarify cost-effectiveness (“it provides value for money”) [15]. The quality of evidence that decisionmakers use can only be determined when several concepts are considered, such as scientific validity, completeness and relevance to the decisionmaking context [18]. The strength of evidence builds with time as interventions are used in real life and initial decisions made in a context of uncertainty (e.g., randomized clinical trial data in limited populations) may be revisited as evidence accumulates. A common question is how much evidence is enough to make an evidence-based decision [59]. Beyond scientific evidence, decisionmaking also relies on colloquial evidence [72]. Consideration of strength and quality of the different types of evidence remain an important part of the appraisal of interventions.

Safety, a critical element of policy and clinical practice, was the sixth most cited criterion. Safety refers to the frequency and severity of adverse events or complications arising as a result of using the new technology compared to an alternative [22]. Efficacy and safety are the main criteria in the initial evaluation of a new intervention [70]. And the risk-to-benefit equation is a critical component of clinical and regulatory decisionmaking [67].

A number of other criteria were identified highlighting the complexity of healthcare decisionmaking and the need to support this process with tools to ensure consistency, transparency and accountability for reasonableness. An important milestone towards that goal would be to harmonize terminology. Indeed, a large variety of terminology was found in the literature during analysis and classification of criteria. Although a systematic approach was used to classify terms into criteria and overarching categories using the principles of MCDA, such analyses are limited by the subjective interpretation of terms reported by authors. For example, the terms reported in published studies such as “side effects,” “unintended consequences,” “risks,” “harm,” or “adverse effects” were all grouped under the criterion “Safety.” These variations of terminology underline the difficulty of harmonizing the decisionmaking processes, as several authors have noted [10,11]. It calls for well-defined criteria to avoid confusion and ensure sound application of multicriteria approaches to decisionmaking [11,73].

Although this analysis was limited to published studies, an extensive analysis of decisionmaking processes from jurisdictions around the world for coverage of healthcare interventions was performed to define the criteria of the EVIDEM framework, which are included in this analysis [10,18]. In addition, the large number of terms retrieved covers criteria currently used in more than 25 decisionmaking processes for coverage of medicines [65].

## Conclusion

This study highlights the importance of considering both normative and feasibility criteria for decisionmaking and priority setting of healthcare interventions. By providing a comprehensive classification of decisionmaking criteria, this analysis can promote reflection on the value of harmonizing terminology in this field. It can also serve as a resource when considering which criteria to include in sound multicriteria approaches (i.e., fulfilling principles of completeness, lack of redundancy, mutual independence, operationalizability and clustering). This analysis is also used as a foundation for the development of an international survey on criteria expected to further expand our knowledge of real-life decisionmaking and advance multicriteria approaches.

Such approaches have the potential to integrate and facilitate pragmatic operationalization of a large range of considerations, including ethical considerations, in a transparent and consistent process. They could provide a common metric for curative and preventive interventions to clearly define best health improvements within resource available, as recently advocated by Volp and colleagues [74]. They may also provide a road map to develop more participative decisionmaking processes by “better combining of many elements” proposed by Culyer [75].

## Competing interests

The author(s) declare that they have no competing interests.

## Authors’ contributions

LAG, MMG, and MW designed the study, collected and analyzed the data and drafted the manuscript. RB, DR, JVT and PK contributed to the design and analyses and reviewed the manuscript. All authors read and approved the final manuscript.
